# The natural history of adolescent idiopathic scoliosis

**DOI:** 10.4103/0019-5413.58601

**Published:** 2010

**Authors:** Hee-Kit Wong, Ken-Jin Tan

**Affiliations:** University Spine Center, University Orthopedics, Hand and Reconstructive Microsurgery Cluster, National University Health System, Singapore

**Keywords:** Adolescent idiopathic scoliosis, curve progression, natural history

## Abstract

There have been great advances in the conservative and surgical treatment for adolescent idiopathic scoliosis in the last few decades. The challenge for the physician is the decision for the optimal time to institute therapy for the individual child. This makes an understanding of the natural history and risk factors for curve progression of significant importance. Reported rates of curve progression vary from 1.6% for skeletally mature children with a small curve magnitude to 68% for skeletally immature children with larger curve magnitudes. Although the patient's age at presentation, the Risser sign, the patient's menarchal status and the magnitude of the curve have been described as risk factors for curve progression, there is evidence that the absolute curve magnitude at presentation may be most predictive of progression in the long term. A curve magnitude of 25° at presentation may be predictive of a greater risk of curve progression. Advances in research may unlock novel predictive factors, which are based on the underlying pathogenesis of this disorder.

## INTRODUCTION

Adolescent idiopathic scoliosis (AIS) is defined by the Scoliosis Research Society as scoliosis whose onset occurs after ten years of age and whose cause is essentially unknown. It is a relatively common condition among adolescents.

AIS has been described as having a prevalence of about 0.35 to 13%, depending on the defined Cobb's angles, screening age and sex.^1^–^3^ Stirling *et al.*, in a school screening study, reported prevalence rates of 0.4 and 2.2% in English girls of 9 to 11 years of age and 12 to 14 years of age, respectively. The boys had prevalence rates of 0.1 and 0.3% in the two age groups.^4^

Soucacos *et al*. reported on the results of screening of 82,900 Greek schoolchildren of 9 to 14 years old during a 1-year prospective study. He reported a prevalence of 1.7% in his study. In total, 2.6% of the girls and 0.9% of the boys had radiographic evidence of a Cobb's angle of 10° or more. He also described the prevalence to vary according to age. A total of 0.07% of the children had scoliosis by the age of 9, 0.2% by age 10 and 0.4% at age 14.^5^

Wong *et al.*, reporting on the prevalence of idiopathic scoliosis in Singaporean school children, found that 0.04% of the children had scoliosis by the age of 7, 0.19% by the age of 10 and 1.44% by the age of 14, while the overall predicted prevalence rate for children 9 to 14 years of age (comprising 9- to 10-, 11- to 12-, 13- to 14-year-old groups) was 0.78% (1.23% in girls and 0.33% in boys).^6^

## LONG-TERM COMPLICATIONS OF UNTREATED SCOLIOSIS

If left undetected and untreated, AIS can lead to many potential complications. Nachemson,^7^ Nilsonne and Lundgren,^8^ Pehrsson *et al*.^9^ and Fowles *et al*.^10^ described a poor prognosis for untreated scoliosis with increased mortality rates related to cor pulmonale and back pain, increased disability and socioeconomic effects on work and marital status. In Nachemson's study, he reported on the outcome of a 38-year follow-up of 130 patients with untreated scoliosis. About 38% were disabled due to their deformity and the mortality rate was 100% above that of the normal population. About 37% had constant backache and 14% complained of cardiopulmonary symptoms. Nilsonne and Lundgren, reporting on a 50-year follow-up of 113 patients, also reported a mortality rate twice that of the general population. Of the remaining patients, half were unable to work, 90% had back pain and 30% were on disability pensions for back pain or scoliosis. However, it should be noted that these studies included patients with other causes of scoliosis, and a significant number of cases of idiopathic scoliosis were of infantile and juvenile types. Therefore, the conclusions may not completely apply to children with AIS who may have a more benign long-term natural history.

Hence, the natural history of AIS in the long-term would be better analyzed in studies that only include patients who have AIS. Weinstein and Ponseti reported on the long-term outcome of a group of 194 patients with untreated AIS. The patients were an average of 53 years of age and out of the 194, all but 4 were normally active. About 21% had mild psychological reactions to their deformity, such as unwillingness to wear tight fitting clothing or a bathing suit. Backache was described as being somewhat more common compared to a matched group of 100 patients but was not disabling. They did not find an increased mortality rate compared to a match group and cor pulmonale was implicated as a cause of death only in one patient.^11^

## SCHOOL-BASED SCREENING

There have been significant improvements in both the conservative as well as operative treatment of AIS in the last few decades. However, it has become apparent that despite these advances, the success of treatment still depends on early detection of this condition. Both conservative treatments as well as spinal instrumentation have the best results before the curve has progressed to a large magnitude.

This has been an important reason behind the institution of screening programs in many countries that allow for the early detection of curves of smaller magnitude.^1^–^3^,^6^ However, this presents the treating physician with the challenge of predicting the risk of curve progression and deciding on appropriate management as well as follow-up intervals and duration for this group of patients.

## CURVE PROGRESSION DURING SKELETAL GROWTH

The decision on the optimal time to institute bracing or surgical treatment as well as when observation of the curve alone is sufficient can only be confidently made with an understanding of the natural history of adolescent idiopathic scoliosis. However, accurate prediction of curve progression is still not available. Reported rates of curve progression have varied considerably [Table 1]. There is also no agreement on the associated risk factors. Some of the factors quoted by previous studies include magnitude of the curve, the patient's age at presentation, the Risser sign and the patient's menarchal status.

**Table 1:** Comparison of inclusion criteria, definition of progression and progression rates reported by studies on curve progression on idiopathic scoliosis

Authors	Number of children	Inclusion criteria (Cobb's angle)	Definition of progression	Progression rate (%)
Brooks *et al.*	474	5° or more	Average of 7°	5
Soucacos *et al*.	839	More than 10°	5° or more	14.7
Rougala *et al*.	603	6° or more	5° or more	6.8

This is compounded by the issue of differences in the criteria for progression, the institution of treatment during follow-up and the length of follow-up. As a result, it is not clear to what extent these factors may be used to accurately predict the natural history of AIS. To date, there has been no agreement and definitive guidelines have yet to be established in the prediction of curve progression and when to institute treatment for each child.

Indeed, it has been reported that a significant number of idiopathic curves may actually improve during the follow-up period. Brooks *et al.*, who studied 474 children with AIS defined at a Cobb's angle of 5° or more, reported that spontaneous improvement was observed in approximately 22% of those on follow-up for an average of one year. In the same study, he reported only a 5% incidence of progression of an average of 7°. There was also no difference in the age, gender or curve location between those that improved or progressed.^12^

Soucacos *et al*. reported a 14.7% incidence of curve progression defined as an increase in Cobb's angle of 5° or more. The authors also reported that an unexpected 9.5% of children in his study showed complete spontaneous resolution of the scoliotic curve, while over 35% of the patients with a left thoracic or left and right thoracolumbar curves showed a spontaneous decrease in the magnitude of their curve of at least 10°. In this study, the authors found that the factors that were most associated with the natural history of the scoliotic curve were gender, curve pattern and maturity. They found that girls showed a higher incidence of progression overall and that the difference was even more pronounced in curves that progressed between 5 and 10°. In addition, curves that developed before menarche were found to have almost twice as great a risk for progression.^5^

In cases detected through school screening, Rougala *et al*. followed 603 children for at least 2 years and reported a 6.8% progression of 5° or more. In addition, 15.4% of the skeletally immature girls with initial curves of 10° or more progressed but he also observed that there was no progression in 20% of the skeletally immature curves with an initial magnitude of 20° or more.^2^

Lonstein and Carlson retrospectively reviewed the progression of 727 children with idiopathic scoliosis defined by a single radiograph showing scoliosis of 29° or less. Due to the varying definitions of curve progression in previous studies which varied from 5 to 10°, Lonstein and Carlson instead defined curve progression in their study as an initial curve of 19° or less that increased at least 10° with the final magnitude being greater than 20° and an initial curve of between 25 and 29° that increased by 5° or more. They reported a curve progression rate of 23.2% and found that curve magnitude, skeletal immaturity and curve pattern were associated with progression.^13^

The various factors associated with progression have also been combined for more useful model for prediction of curve progression. Lonstein and Carlson used a combination of curve magnitude and the Risser sign to calculate the probability of curve progression. They found that for children with a Risser grade of 0–1, those with a curve magnitude of 5–19° had a 22% progression rate compared to a 68% progression rate for those with a curve magnitude of 20–29°. Similarly, for children with a Risser grade of 2–4, those with a curve magnitude of 20–29° had a progression rate of 23%, while those with a curve magnitude of 5–19° had only a 1.6% progression rate^13^ [Table 2]. Nachemson and Peterson used a combination of age and curve magnitude. They observed that in children of 16 years of age with an initial curve magnitude of less than 19°, none of the curves progressed. However, in children aged 10–12 years with a curve magnitude of 60° there was a 100% progression rate.^14^ [Table 3].

**Table 2:** Probabilities of curve progression based on Risser grade and curve magnitude

Risser grade	Curve magnitude and associated progression rate	
	
	5–19° (%)	20–29° (%)
0–1	22	68
2–4	1.6	23

Adapted from Lonstein JE, Carlson JM. The prediction of curve progression in untreated idiopathic scoliosis during growth. J Bone Joint Surg Am 1984;66:1061–71

**Table 3:** Probabilities of curve progression based on curve magnitude and age

Curve magnitude	Age and associated progression rate		
	
	10–12 years (%)	13–15 years (%)	16 years (%)
<19°	25	10	0
20–29°	60	40	10
30–39°	90	70	30
60°	100	90	70

Adapted from Nachemson AL, Peterson LE. Effectiveness of treatment with a brace in girls who have adolescent idiopathic scoliosis: a prospective, controlled study based on data from the Brace Study of the Scoliosis Research Society. J Bone Joint Surg Am 1995;77:815–822

## GENETIC PROFILING AS A MEANS TO PREDICT CURVE PROGRESSION

There is an increasing evidence that genetic factors have a part to play in the development in idiopathic scoliosis.^15^ Together with new studies that have investigated the role of hormonal factors such as melatonin and estrogens, there has been interest in studying the genetic polymorphisms of these hormones in an attempt to predict curve development and progression.^16^–^18^ However, there is still no clear genetic marker that can be reliably used to predict progression of the curve. Qiu *et al*. performed genetic association studies to investigate variation of the melatonin receptor 1A (MTNR1A) and 1B genes in girls with adolescent idiopathic scoliosis (AIS) patients compared to normal controls. He found an association with the 1B gene but not the 1A gene polymorphism.^16^,^17^ Inoue *et al*. analyzed the estrogen receptor gene in 304 girls with idiopathic scoliosis whom he followed up till skeletal maturity. He found that the Xbal site polymorphism was associated with curve progression defined as an increase of more than 5° from initial evaluation.^18^

More recently, there has been progress in using a combination of multiple genes associated with the development of severe curves to predict curve progression. Ogilvie has developed a saliva-based test for a combination of multiple genetic markers that have been linked with curve progression in clinical trials as a prognostic test for AIS. This allows for the calculation of a quantitative score that correlates with a low, medium or high risk of curve progression.^19^

## CURVE PROGRESSION AFTER SKELETAL MATURITY

Once the child has attained skeletal maturity, it was generally thought that the curves are less likely to progress. However, this may not always be the case. It is now established that curves due to idiopathic scoliosis do not necessarily stop progressing after skeletal maturity. In a long-term follow-up study of patients with idiopathic scoliosis, Collis and Ponseti found that curves of a larger degree did increase after skeletal maturity.^20^ In a separate study with an average follow-up of 40 years, Weinstein and Ponseti also found that a significant number of idiopathic curves increased after skeletal maturity. They reported that in thoracic curves, the Cobb's angle, apical vertebral rotation and the Mehta angle were important prognostic factors. For lumbar curves, the degree of apical vertebral rotation, the Cobb's angle, the direction of the curve and the relationship of the fifth lumbar vertebra to the inter-crest line were of prognostic value. However, they also observed that curves that were less than 30° at skeletal maturity tended not to progress regardless of curve pattern.^21^

Given the varying definitions of curve progression, this suggests that curves with a Cobb's angle of 30° are an important threshold magnitude and may serve as an endpoint for prediction of curve progression rather than predefined units of curve progression quoted in previous studies. In addition, it must be appreciated that the various associated factors and predictions described only apply to the likelihood of a curve progressing in adolescence. They are only averages and correlations and do not allow us to answer the key issue of how much the curve of an individual child is going to progress.

In a recent study, Tan and Wong reported on a group of 279 patients with idiopathic scoliosis detected by school screening, and who were followed-up until skeletal maturity using a 30° Cobb's angle at skeletal maturity as a threshold instead of predefined units of curve progression during shorter periods of growth. They found that an initial Cobb's angle of 25° was the most predictive factor for curve progression to this threshold magnitude. Initial age, gender and pubertal status were less important prognostic factors.^22^ When different factors were combined, it was also possible to generate different risk progression profiles [Table 4].

**Table 4:** Logistic regression table showing the different probabilities of curve progression based on a combination of factors

Gender	Puberty	Initial Cobb's angle ≥25°	Age at presentation <12 years	Probability of final Cobb ≥30° (%)
Female	No	Yes	Yes	82.2
Female	No	Yes	No	79.6
Female	Yes	Yes	Yes	67.0
Male	No	Yes	Yes	64.6
Female	Yes	Yes	No	63.1
Male	No	Yes	No	60.6
Male	Yes	Yes	Yes	44.4
Male	Yes	Yes	No	40.3
Female	No	No	Yes	14.4
Female	No	No	No	12.4
Female	Yes	No	Yes	6.9
Male	No	No	Yes	6.2
Female	Yes	No	No	5.9
Male	No	No	No	5.3
Male	Yes	No	Yes	2.8
Male	Yes	No	No	2.4

Reprinted from Tan KJ, Moe MM, Vaithinathan R, Wong HK. Curve progression in idiopathic scoliosis: follow-up study to skeletal maturity. Spine 2009;34:697-700

## CONCLUSION AND FUTURE PERSPECTIVE

Adolescent idiopathic scoliosis is a relatively common condition that if left untreated can lead to significant morbidity and possibly mortality. Early detection of curves has been facilitated by school-based screening but has resulted in a need for the understanding of the natural history and reliable prediction of curve progression to decide on the appropriate treatment and timing of intervention. Individual factors such as the patient's age at presentation, the Risser sign, the patient's menarchal status and the magnitude of the curve have been used as predictive factors as well as using a combination of factors to predict curve progression. However, the relative importance of each factor and how they may interact is as yet not defined. Based on current evidence, we recommend closer follow-up of skeletally immature children with a curve magnitude of 25° or more at presentation and also continuing to follow-up children with a curve magnitude of 30° or more even after skeletal maturity. Moving forward, the ideal prognostication model needs to be one that allows for prediction of the amount of progression that is likely to be observed over the whole period of remaining growth and should be individualized to the child. This may only be attained by the introduction of novel predictive factors rather than factors that are surrogate markers of potential remaining skeletal growth that have been used in the literature thus far. This may only be realized by unlocking the underlying pathogenesis of AIS.
